# Merkel cell carcinoma with synchronous but spatially distinct squamous cell carcinoma of the head: Case report of differing PRAME expression and response to checkpoint inhibitor therapy

**DOI:** 10.1016/j.jdcr.2025.05.051

**Published:** 2025-07-17

**Authors:** Claire B. Abbott, Sonali Nanda, Minh Phan, Sepideh Asadbeigi, Jeffrey McBride

**Affiliations:** a OU Health Sciences Center, College of Medicine, Oklahoma City, Oklahoma; b Department of Dermatology, OU Health Sciences Center, Oklahoma City, Oklahoma; c Department of Dermatology, Scripps Health, San Diego, California; d Department of Hematology-Oncology, OU Health Sciences Center, Oklahoma City, Oklahoma; e Department of Pathology, OU Health Sciences Center, Oklahoma City, Oklahoma

**Keywords:** Merkel cell carcinoma, pembrolizumab, PRAME, squamous cell carcinoma

## Introduction

Merkel cell carcinoma (MCC) is a rare and aggressive skin cancer most commonly seen in elderly, Caucasian populations with a history of UV radiation exposure or who are immunocompromised.^1^^,^^2^ Approximately 80% of cutaneous MCC are positive for Merkel cell polyomavirus (MCPyV); however, a smaller subset are MCPyV negative, with UV exposure being the predominant risk factor.^1^ For virus-negative MCC, signature UV-induced DNA mutations with high mutational burden are thought to facilitate MCC tumorigenesis similar to squamous cell carcinoma (SCC), including mutations in TP53, Rb, and PIK3CA.^1^ PReferentially Expressed Antigen in Melanoma (PRAME) is a relatively newly utilized marker for cutaneous malignancies; it is a well-characterized leucine-rich repeat cancer-testis antigen within the PRAME family.^1^^,^^2^ It is thought that PRAME plays a role in the malignant phenotype of some cancer cells including melanoma and other cutaneous and noncutaneous malignancies.^1^^,^^2^

Pembrolizumab (Keytruda, Merck & Co, Inc), a T-cell PD-1 inhibitor that prevents the binding of PD-1 to its ligands, programmed cell death ligand 1 and programmed cell death ligand 2, which are frequently overexpressed on cancer cells, is regarded as an efficacious treatment of MCC and SCC and is Food and Drug Administration-approved for the treatment of both malignancies.^3^^,^^4^ Our goal is to deliberate the unique response of co-occurring but anatomically separate MCC and SCC of the head to treatment with pembrolizumab and how PRAME expression plays a role in their respective responses.

## Case report

**Fig 1:**
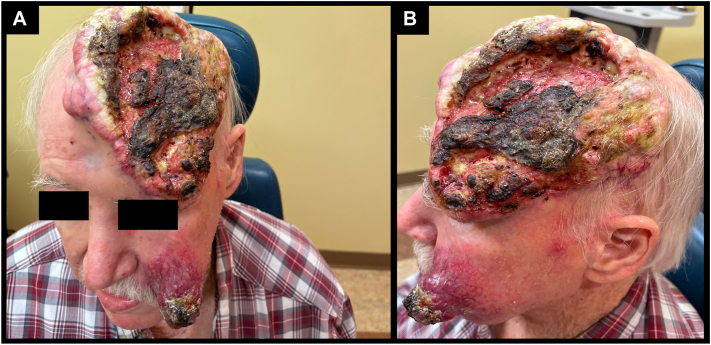
**A** and **B,** Merkel cell carcinoma and squamous cell carcinoma. Clinical images depicting a very large (∼15 cm) ulcerated, exophytic mass covering the entirety of the left frontal scalp (SCC) and a large fungating mass (∼6 cm) on the medial left cheek (MCC) from August 23, 2023. *MCC*, Merkel cell carcinoma; *SCC*, squamous cell carcinoma.

**Fig 2:**
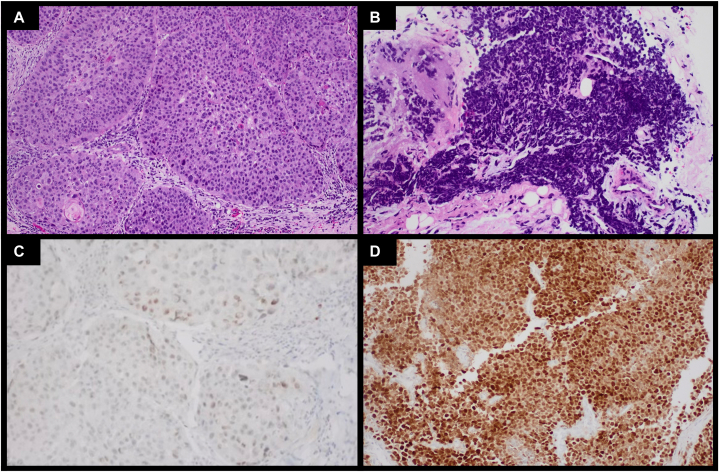
**A,** Squamous cell carcinoma. H&E stain of the scalp lesion at 200× magnification consistent with moderately differentiated squamous cell carcinoma. **B,** Merkel cell carcinoma. H&E stain of the left cheek lesion at 200× magnification consistent with Merkel cell carcinoma. **C,** Squamous cell carcinoma. PRAME staining of the squamous cell carcinoma at 200× magnification shows low intensity, low-expressing, variable PRAME staining highlighting <25% of cells. **D,** Merkel cell carcinoma. PRAME staining of the Merkel cell carcinoma at 200× magnification shows diffuse positivity for PRAME with strong intensity in >95% of cells. *PRAME*, PReferentially Expressed Antigen in Melanoma.

A 78-year-old Caucasian male presented with a large left scalp lesion that had been growing over the last year and a large fungating left medial cheek mass that had been growing for 7 months (Fig 1). A scalp biopsy was obtained for histopathological evaluation, which was consistent with SCC, and a month later, a left cheek biopsy with immunohistochemistry targeting MCPyV antigen was consistent with virus-negative MCC (Fig 2). On immunohistochemistry, the MCC was diffusely high-expressing for PRAME with strong intensity in >95% of cells. In contrast to the MCC, PRAME expression in the SCC was minimal, with <25% of cells and only light intensity (Fig 2). A staging positron emission tomography/computed tomography scan was significant for a 15-centimeter hypermetabolic soft tissue thickening along the left frontal scalp with multifocal invasion of the frontal bone and a 5.7-centimeter hypermetabolic left cheek mass. In addition to the imaging showing no suspicious lymph node activity, the patient was a poor surgical candidate; therefore, no lymph node dissection was pursued. The case was discussed at the head and neck tumor board, and due to the extent of the disease, he was again deemed a poor surgical candidate. He then established care with medical oncology to discuss treatment with palliative immune checkpoint inhibitor therapy.

**Fig 3:**
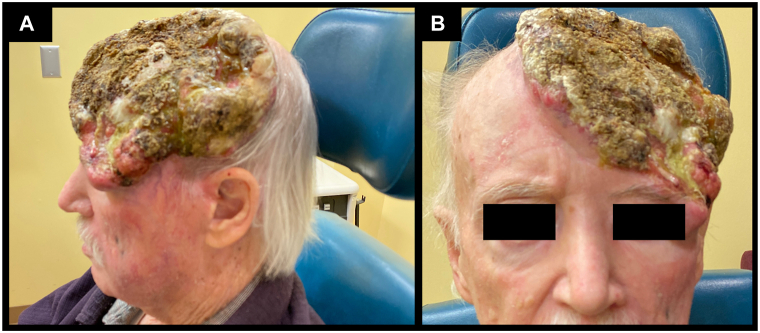
**A** and **B,** Merkel cell carcinoma and squamous cell carcinoma. Clinical images grossly depicting the pembrolizumab-unresponsive scalp SCC and the clinical resolution of the MCC on the left cheek. *MCC*, Merkel cell carcinoma; *SCC*, squamous cell carcinoma.

The patient began immunotherapy with monthly intravenous pembrolizumab infusions for a total of 6 cycles to which the MCC responded clinically, but the SCC continued to invade surrounding tissues (Fig 3). A computed tomography head after 4 months of treatment showed significant improvement in the cheek MCC with only residual edema and inflammatory changes to surrounding adipose tissue, whereas the scalp SCC showed worsening erosion through the left frontal calvarium, an increase in underlying dural thickening, enhancement with bony erosion through the superolateral aspect of the orbit, and extension to the extraconal space with mild mass effect on the orbit.

Current plans are for the patient to undergo next-line treatment with the cytotoxic chemotherapy carboplatin and paclitaxel in replacement of pembrolizumab and to meet with radiation oncology to address the pembrolizumab-unresponsive SCC. Long-term follow-up for the clinically resolved MCC includes monthly physical examinations and computed tomography imaging every 3 months to assess the SCC treatment response and to monitor for MCC recurrence.

## Discussion

This case illustrates an extraordinary co-occurrence of PRAME-high-expressing MCC with PRAME-low-expressing moderately differentiated SCC and suggests that PRAME expression may be a predictor of the divergent response of the tumors to initial treatment with pembrolizumab. PRAME expression in MCC has been shown to have variable expression and can exhibit high intensity and diffuse PRAME expression or no expression at all.^5^^,^^6^ SCC has been noted to display no to low intensity PRAME expression but has also been found to show focal high intensity expression with random positivity of junctional melanocytes in some cases.^6^^,^^7^ This pattern of PRAME expression in SCC suggests an association with sun exposure and can have implications for the assessment of sample margins in chronically sun-damaged skin.^6^ In addition, upregulation of PRAME expression in SCC is often associated with a more aggressive phenotype.^8^

There has been limited research regarding PRAME as a potential target for treatment of malignancy and how PRAME expression influences treatment response. PRAME, however, is emerging as a promising immunologic target, and PRAME-specific T-cell-based immunotherapy and PRAME-targeted vaccines are currently being explored.^6^ The prospect of utilizing PRAME-targeted therapies in combination with immunotherapeutic agents holds potential for a synergistic effect.^6^ Studies have indicated that elevated PRAME expression correlates with advanced disease stages and poorer survival outcomes,^6^ in addition to the association of higher PRAME levels with the overexpression of ligands associated with immunoregulation.^2^ These ligands, such as programmed cell death ligand 1, CD86, GAL-9, and VISTA, may partly explain the reduced T-cell-mediated activation and killing of PRAME-overexpressing cancer cells.^2^ This may also suggest an immunoregulatory role for PRAME, further indicating the utility of PRAME-based therapy in combination with immunotherapy.^2^

This case highlights the potential prognostic and therapeutic significance of PRAME expression in cutaneous malignancies. The divergent responses of PRAME-high-expressing MCC and PRAME-low-expressing SCC to pembrolizumab emphasize the importance of understanding PRAME’s role in tumor biology and treatment outcomes. While PRAME expression appears to correlate with responsiveness to immunotherapy in MCC, its absence in SCC suggests a lack of immunologic vulnerability, potentially explaining the SCC’s resistance to pembrolizumab. A large-scale study to examine the responses of keratinocyte carcinomas with differences in PRAME expression to immunotherapy will be necessary to further examine this divergence in response to treatment. In addition, further investigation into PRAME as a biomarker and therapeutic target may refine treatment strategies, offering the possibility of personalized therapy based on PRAME expression. As PRAME-targeted therapies continue to be explored, future research should focus on the implications of PRAME expression in treatment-resistant malignancies, providing insight into more effective combinations of immunotherapeutic agents.

## Conflicts of interest

None disclosed.
